# Gait speed as predictor of transition into cognitive impairment: Findings from three longitudinal studies on aging

**DOI:** 10.1016/j.exger.2019.110783

**Published:** 2019-11-18

**Authors:** Emiel O. Hoogendijk, Judith J.M. Rijnhart, Johan Skoog, Annie Robitaille, Ardo van den Hout, Luigi Ferrucci, Martijn Huisman, Ingmar Skoog, Andrea M. Piccinin, Scott M. Hofer, Graciela Muniz Terrera

**Affiliations:** a Department of Epidemiology and Biostatistics, Amsterdam Public Health Research Institute, Amsterdam UMC - location VU University Medical Center, Amsterdam, the Netherlands; b Department of Psychology, Centre for Health and Ageing AGECAP, University of Gothenburg, Gothenburg, Sweden; c Département de Psychologie, Université du Québec, Montréal, QC, Canada; d Department of Statistical Science, University College London, London, UK; e National Institute on Aging, Baltimore, MD, USA; f Department of Sociology, VU University, Amsterdam, the Netherlands; g Institute of Neuroscience and Physiology, Centre for Health and Ageing AGECAP, University of Gothenburg, Gothenburg, Sweden; h Department of Psychology, University of Victoria, Victoria, BC, Canada; i Centre for Dementia Prevention, The University of Edinburgh, Edinburgh, UK

**Keywords:** Cognition, Dementia, Walking speed, Multistate modeling

## Abstract

**Objectives:**

Very few studies looking at slow gait speed as early marker of cognitive decline investigated the competing risk of death. The current study examines associations between slow gait speed and transitions between cognitive states and death in later life.

**Methods:**

We performed a coordinated analysis of three longitudinal studies with 9 to 25 years of follow-up. Data were used from older adults participating in H70 (Sweden; n = 441; aged ≥70 years), InCHIANTI (Italy; n = 955; aged ≥65 years), and LASA (the Netherlands; n = 2824; aged ≥55 years). Cognitive states were distinguished using the Mini-Mental State Examination. Slow gait speed was defined as the lowest sex-specific quintile at baseline. Multistate models were performed, adjusted for age, sex and education.

**Results:**

Most effect estimates pointed in the same direction, with slow gait speed predicting forward transitions. In two cohort studies, slow gait speed predicted transitioning from mild to severe cognitive impairment (InCHIANTI: HR = 2.08, 95%CI = 1.40–3.07; LASA: HR = 1.33, 95%CI = 1.01–1.75) and transitioning from a cognitively healthy state to death (H70: HR = 3.30, 95%CI = 1.74–6.28; LASA: HR = 1.70, 95%CI = 1.30–2.21).

**Conclusions:**

Screening for slow gait speed may be useful for identifying older adults at risk of adverse outcomes such as cognitive decline and death. However, once in the stage of more advanced cognitive impairment, slow gait speed does not seem to predict transitioning to death anymore.

## 1. Introduction

In aging societies, the growing number of people with dementia comprises a major challenge for sustainability of healthcare systems (Shah et al., 2016). There is also much individual burden for a person living with dementia, such as loss of healthy life years (Murray et al., 2013). It is therefore paramount to better understand the cognitive aging process, and to increase our insights into risk factors for transitioning from a cognitively healthy state to cognitive impairment.

Recently, there has been an increasing interest in slowing gait speed as early indicator of cognitive impairment (Rosano and Snitz, 2018; Peel et al., 2019). The idea is that slowing gait may be related to cognitive decline through various underlying mechanisms, such as neurodegeneration, inflammation, and physical inactivity (Hackett et al., 2018; Quan et al., 2017). It has also been suggested that slowing gait speed is an expression of motor decline in early dementia stages, as emphasized by recent literature on predementia syndromes such as the motoric cognitive risk syndrome (Verghese et al., 2014). Evidence comes from cohort studies that demonstrate longitudinal associations between slow gait speed and cognitive decline or dementia (Quan et al., 2017), but also from brain imaging studies that show associations between Alzheimer’s disease pathology and decreased gait speed (Del Campo et al., 2016). However, very few studies looking at slow gait speed as early marker of cognitive decline have investigated the competing risk of death. The relationship between gait speed and mortality in older adults is well-established (Studenski et al., 2011). Therefore, not taking into account the competing risk of death may result in overestimation of the effect of slow gait speed on cognitive decline.

Multistate models may be used to study transitions across health states and death simultaneously and to study the effect of risk factors on state transitions. To our knowledge, multistate modelling (MSM) has not been applied to study the role of gait speed in the prediction of transitions across cognitive states and death. Therefore, the aim of this study was to examine associations between slow gait speed and transitions between cognitive states and death in later life. This was done through a coordinated MSM analysis using data from three longitudinal studies on aging.

## 2. Methods

### 2.1. Study populations

For this analysis, we used data from three longitudinal studies. See below for a brief description of each study. We included participants with valid baseline data on the Mini-Mental State Examination (MMSE), information on covariates (age, sex, education and gait speed), and at least two known states (MMSE state or death state). The three studies were conducted in line with the Declaration of Helsinki, and were approved by local medical ethics committees. All respondents or their legal representatives provided written informed consent.

#### 2.1.1. H70

The H70 studies are multidisciplinary epidemiological cohort studies in older populations in Gothenburg, Sweden. Various cohorts with a baseline age of 70 have been followed over time. The H70 studies have been described in detail before (Rydberg Sterner et al., 2019; Skoog et al., 2019). In the current study, we included data from the cohort born in 1930, with four measurement waves at ages 70, 75, 79, and 85 years. Of 604 participants at baseline, 441 respondents met the inclusion criteria, who provided 1463 state observations.

#### 2.1.2. InCHIANTI

The Invecchiare in Chianti, aging in the Chianti area (InCHIANTI) study is an ongoing longitudinal study from Italy that focuses on mobility decline and related factors in later life. Details on the sampling and design have been published before (Ferrucci et al., 2000). In the current study, we included data of people aged 65 and over at baseline (1998–2000) and three follow-up waves (2001–2003, 2004–2006, and 2007–2009). Of the 1155 participants at baseline, we included 955 respondents with valid data in the final sample, who provided 3386 state observations.

#### 2.1.3. LASA

The Longitudinal Aging Study Amsterdam (LASA) is an ongoing, multidisciplinary study among a representative sample of older adults aged 55 year and over in the Netherlands. Details on the LASA sampling and methods have been published previously (Hoogendijk et al., 2016). In the current study, data from baseline in 1992–1993 and six follow-up waves (1995–1996, 1998–1999, 2001–2002, 2005–2006, 2008–2009, and 2011–2012) were included. Of 3107 participants at baseline, there were 2824 respondents with valid data, who provided 12655 state observations.

### 2.2. Measures

#### 2.2.1. Cognitive functioning

We used MMSE to assess global cognitive functioning at baseline and at follow-up measurement waves (Folstein et al., 1975). As in a previous publication, three states were distinguished: normal cognition (MMSE 27–30), mild impairment (MMSE 23–26), and severe impairment (MMSE ≤22) (Robitaille et al., 2018).

#### 2.2.2. Mortality

Mortality status, including date of death, was retrieved from municipality registers (InCHIANTI, LASA) or from a national death registration system (H70).

#### 2.2.3. Covariates

Covariates in the analyses included age, sex, education (years), and slow gait speed. All covariates were measured at baseline, except for age (included as a time-varying variable). Gait speed assessment included maximum gait speed for 30-m indoors (H70), usual gait speed for 4-m (InCHIANTI), and gait speed for 6-m with turn at 3-m (LASA). Since gait speed measures varied across studies, it was not possible to use established cut-points for slow gait. Therefore, we applied the lowest quintile approach (sex-specific), which is validated in many frailty studies (Saum et al., 2012). Cut-points for H70 were ≤1.77 m/s for men and ≤1.55 m/s for women, for InCHIANTI ≤0.93 m/s for men and ≤0.75 m/s for women, and in LASA ≤0.60 m/s for both men and women.

### 2.3. Statistical analysis

We used a coordinated analysis approach, which implies running consistent analytical models across independent studies, using the same variables. MSM was used to assess transitions between cognitive states and death. These models allow for simultaneously analyzing transitions between health states and examining the association of covariates with these transitions. Because of the Markov assumption underlying MSM, the information that is used to estimate the model parameters is based on pairs of consecutive observations only. Therefore, with sufficient transition data in each of the three included cohorts, no bias will be introduced when there is cohort variation in the length of follow-up (i.e., the number of follow-up measurements).

**Fig. 1.**
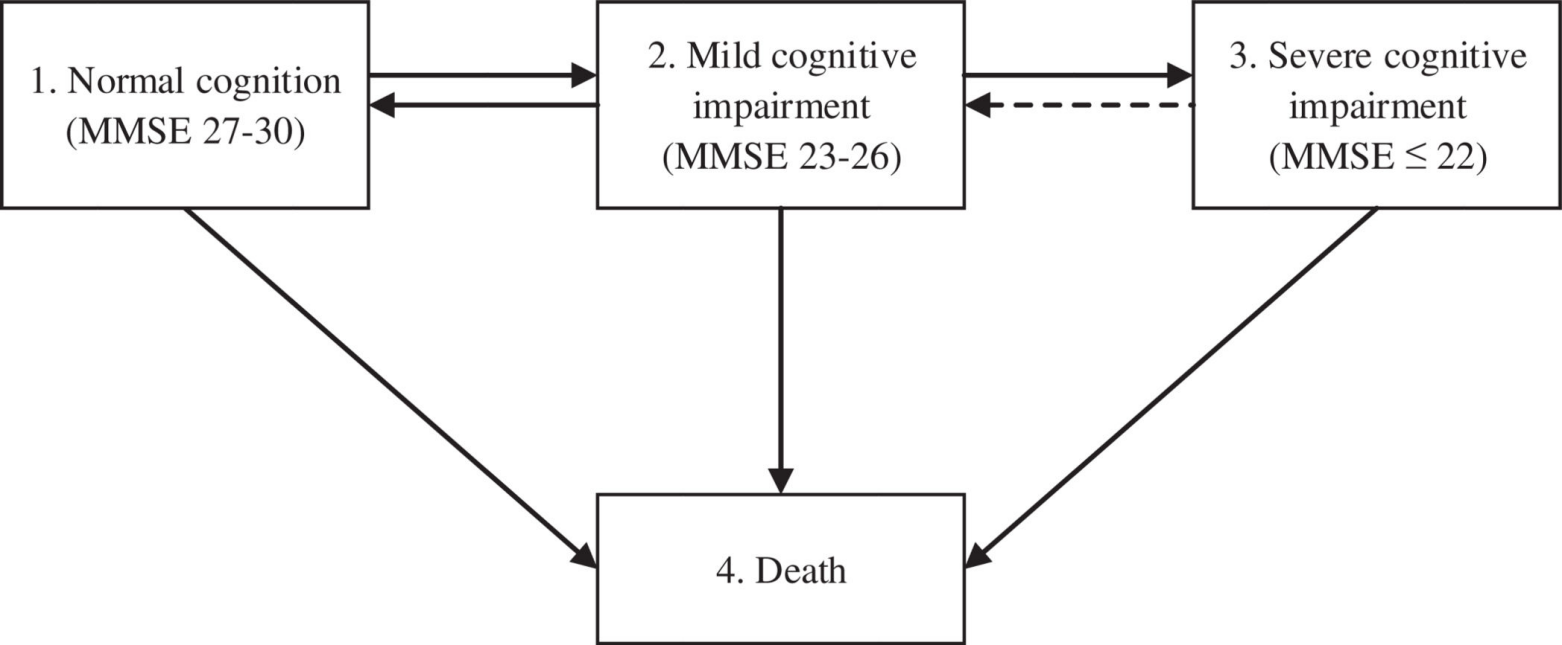
Design: four-state model.

A four-state model was applied: state 1 was normal cognition, state 2 mild cognitive impairment, state 3 severe cognitive impairment, and state 4 was death as the absorbing state (Fig. 1). Age, sex, education, and slow gait speed were included as covariates on all transitions modeled, except for gait speed in H70, that was excluded from the transition from state 2 to state 4 due to the low number of cases. We allowed backward transitions from mild cognitive impairment (state 2) to normal cognition (state 1), and included a misclassification model for InCHIANTI and LASA. This means that individuals were not allowed to transition from state 3 to state 2 without it being a result of a misclassification. The estimated misclassification probabilities were low (InCHIANTI = 0.18; LASA = 0.10). Hazard ratios and 95% confidence intervals for state transitions were provided for slow gait speed. Multistate models were estimated using the MSM package in R (Jackson, 2011).

## 3. Results

Table 1 shows the baseline characteristics for each cohort study. The mean age ranged from 70.3 years (LASA) to 75 years (InCHIANTI), and across all studies the majority of the sample was female. At baseline, no cognitive impairment was observed in 89% (H70), 42% (InCHIANTI), and 69.9% (LASA) of the respondents.

The results of the multistate models for the effect of slow gait speed are presented in Table 2, all adjusted for age, sex and educational level. In two studies, statistically significant associations were found between slow gait speed and transitioning from a cognitively healthy state to death (H70: HR = 3.30, 95% CI = 1.74–6.28; LASA: HR = 1.70, 95% CI = 1.30–2.21). Also in two studies, slow gait speed was associated with a higher risk of transitioning from mild to severe cognitive impairment (InCHIANTI: HR = 2.08, 95% CI = 1.40–3.07; LASA: HR = 1.33, 95% CI = 1.01–1.75). Only in the LASA study, slow gait speed was associated with a higher risk of transitioning from normal cognition to mild cognitive impairment (HR = 1.23, 95% CI = 1.00–1.50), and with transitions between mild cognitive impairment and death (HR = 1.56, 95% CI = 1.06–2.29). Slow gait speed did not predict transitioning from severe cognitive impairment to death (state 3 to state 4) in any of the studies. The effects of slow gait speed on backward transitions from mild cognitive impairment to normal cognition were not statistically significant, but all pointed in the same direction (i.e., those with slow gait speed were less likely to transition from state 2 to state 1).

The effects of age and sex (results not shown) in the multistate models were as expected across all studies: a higher age is associated with forward transitions, and males are more likely to die sooner.

## 4. Discussion

In this coordinated analysis of three longitudinal studies on aging, we examined associations between slow gait speed and transitions between cognitive states and death. We found in two studies that slow gait speed predicted transitioning from mild to severe cognitive impairment. Our results also revealed that slow gait speed is associated with mortality, but mainly in people without cognitive impairment. These results suggest that screening for slow gait speed may be useful for identifying older adults at risk of adverse outcomes such as cognitive decline and death, but only when older adults are still cognitively healthy, or at an early stage of cognitive impairment.

The increasing interest in slow gait speed as an early indicator of cognitive decline is understandable (Peel et al., 2019; Hackett et al., 2018; Quan et al., 2017; Best et al., 2016; Montero-Odasso et al., 2018; Beauchet et al., 2016). Gait speed is easy to measure, and there are plausible links between cognition and motor functioning (Rosano and Snitz, 2018). Previous studies on the associations between slowing gait and cognitive decline or dementia observed similar results compared to our study. One study observed that a decline in gait speed precedes cognitive decline (Best et al., 2016). Another study found that slowing gait may already be present 12 years prior to the development of mild cognitive impairment (Buracchio et al., 2010). Previous studies did, however, not take into account the competing risk of death (Peel et al., 2019; Quan et al., 2017).

The finding that slow gait speed was not related to transitioning to death in persons with more advanced cognitive impairment is novel, and suggests that slow gait speed is not the most useful tool to identify older adults at risk when cognitive decline already has started. A possible explanation for this finding is that at a more advanced stage of cognitive impairment, associated comorbidities or the cognitive impairment itself play a more important role in the prediction of death than indicators of motor function such as slow gait speed. Further research is needed on the underlying causes and mechanisms of age-related decline in gait speed, and the extent to which these mechanisms differ when it concerns the prediction of cognitive decline or death.

This was one of the first studies to investigate the impact of slow gait speed on cognition and death simultaneously. Strengths of the study include the coordinated analysis that allowed for replication of results across several studies, the inclusion of a large number of state observations from three cohort studies on aging, and the robust analytical approach (MSM). Although the three included cohort studies differed substantially in terms of sample size, gait speed measures and baseline distributions in cognitive functioning, most effect estimates pointed in the same direction. Therefore, our study provides a better insight into generalizability of results compared to studies that only include data from one sample.

The study also has some limitations. First, we used the MMSE to determine cognitive status, but it should be noted that the MMSE only gives an indication of mild cognitive impairment and dementia, and that it is not the same as clinical diagnosis. Second, we assessed gait speed only at baseline. Since previous research has shown that the trajectory of gait speed preceding cognitive decline is important to consider, time-varying gait speed measures may be considered in future MSM studies (Buracchio et al., 2010). Unfortunately, we did not have time-varying gait speed measures available in all of the cohorts included in the current study. However, at the same time, gait speed measurement at one time-point may better align with the clinical setting, where it is often not feasible to track gait speed changes of patients over an extended time period. Third, we adjusted the analyses for age, sex and educational level, but we did not control for additional health-related factors such as disability and multi-morbidity. This could be explored in future research. Finally, gait speed measures differed across studies. For the current study, this was overcome by applying the lowest quintile approach to define slow gait (Saum et al., 2012). However, a more standardized approach would be needed to obtain cut-points that could be used in clinical practice.

Eventually, findings from this study – and other studies focused on motor function and cognitive decline - may be used to inform clinical practice, and to develop interventions focused on delaying or preventing cognitive decline. Screening for slow gait speed could identify older adults at an early and potentially reversible stage of the process of cognitive decline. An additional advantage of screening for slow gait speed is that it predicts various other adverse health outcomes as well, which could be intervened on (Abellan van Kan et al., 2009). The question is whether gait speed loss that is associated with cognitive decline can be prevented, or whether interventions focused on maintaining gait speed can slow down cognitive decline. There is, for example, a lack of firm evidence base for physical activity interventions that aim to delay or prevent cognitive decline in later life (Brasure et al., 2018). More robust evidence from long-term clinical trials is needed.

## 5. Conclusions

This coordinated analysis provided partial evidence for the role of slow gait speed as early indicator of cognitive decline and death. In two out of three studies, slow gait speed was a predictor of transitioning from mild to severe cognitive impairment, as well as a predictor of the transition to death in cognitively healthy people. However, once in the stage of advanced cognitive impairment, slow gait speed does not seem to predict transitioning to death anymore.

## Figures and Tables

**Table 1:** Baseline characteristics for each study.

	H70	InCHIANTI	LASA
	
	(n = 441)	(n = 955)	(n = 2824)
Country	Sweden	Italy	The Netherlands
Age, mean (SD)	70.6 (0.66)	75.0 (7.1)	70.3 (8.7)
Sex (female), n (%)	247 (56.0)	527 (55.2)	1447 (51.2)
Education (years), mean (SD)	10.3 (4.5)	5.4 (3.3)	8.8 (3.3)
Gait speed (m/s), mean (SD)	1.9 (0.4)	1.03 (0.3)	0.82 (0.3)
Gait speed^a^			
Slow, n (%)	94 (21.3)	198 (20.7)	595 (21.1)
Normal, n (%)	347 (78.7)	757 (79.3)	2229 (78.9)
MMSE – n (%)			
No impairment	393 (89.1)	401 (42.0)	1975 (69.9)
Mild impairment	46 (10.4)	371 (38.8)	690 (24.4)
Moderate to severe impairment	2 (0.5)	183 (19.2)	159 (5.6)

a Slow gait, based on lowest quintile, sex-specific.

**Table 2:** Hazard ratios and 95% confidence intervals for the effect of slow gait speed on state transitions.

Transitions	Slow gait speed (lowest quintile, sex specific)		
	H70	InCHIANTI	LASA
	
	HR (95% CI)	HR (95% CI)	HR (95% CI)
State 1 – State 2	1.36 (0.70–2.68)	1.00 (0.63–1.60)	1.23 (1.00–1.50)*
State 1 – State 4	3.30 (1.74–6.28)*	1.68 (0.61–4.61)	1.70 (1.30–2.21)*
State 2 – State 1	0.85 (0.29–2.48)	0.71 (0.42–1.20)	0.85 (0.65–1.10)
State 2 – State 3	1.06 (0.49–2.28)	2.08 (1.40–3.07)*	1.33 (1.01–1.75)*
State 2 – State 4	–	0.97 (0.19–5.09)	1.56 (1.06–2.29)*
State 3 – State 4	0.56 (0.24–1.30)	1.39 (0.97–1.97)	1.12 (0.91–1.37)

All multistate models contained age, sex and educational level as covariates; State 1 = normal cognition, State 2 = mild cognitive impairment, State 3 = severe cognitive impairment, State 4 = death; HR = hazard ratio, 95% CI = 95% confidence interval.

* Statistically significant hazard ratio (p < .05).
